# Parasitic Contamination of Fruits and Vegetables Collected from Selected Local Markets of Jimma Town, Southwest Ethiopia

**DOI:** 10.1155/2014/382715

**Published:** 2014-08-10

**Authors:** Tamirat Tefera, Abdissa Biruksew, Zeleke Mekonnen, Teferi Eshetu

**Affiliations:** Department of Medical Laboratory Sciences and Pathology, College of Public Health and Medical Sciences, Jimma University, 378 Jimma, Ethiopia

## Abstract

*Background*. A study aimed at determining the prevalence and predictors of parasitic contamination of fruits and vegetables collected from local markets in Jimma Town, Ethiopia, was conducted between April and May 2013. *Methods*. A total of 360 samples of fruits and vegetables were examined by sedimentation concentration after washing using normal saline. *Results*. The overall prevalence of parasitic contamination was 57.8%. *Strongyloides* like parasite (21.9%) was the most frequent parasitic contaminant followed by *Toxocara Spp* (14.7%), *Cryptosporidium Spp* (12.8%), *H. nana* (8.3%), *G. lamblia* (7.5%), *A. lumbricoides* (6.7%), *E. histolytica/dispar* (5.3%), *Cyclospora spp* (5.0%), and *H. diminuta* (1.4%). Washing of the fruits and vegetables before display for selling was significantly associated with decreased parasitic contamination (*P* < 0.001). *Conclusion*. Since fruits and vegetables are potential sources of transmission for intestinal parasites in the study area, consumers should always avoid acquiring parasitic infection from contaminated fruits and vegetables supplied in Jimma Town through proper cleaning and cooking.

## 1. Introduction

Intestinal parasitic infections are widely distributed throughout the world causing substantial intimidation to the public health, economy, and physical and cognitive development particularly among children in developing countries like Ethiopia. The poor personal hygiene, poor environmental hygiene, and poor health system commonly observed in developing countries make the prevalence to be highest among these populations [1, 2].

The consumption of fruits and vegetables helps in protecting human body from a number of diseases by providing nutrients, vitamins, minerals, protein, and fibers. It could also have a positive impact on body-weight regulation and related conditions, including diabetes and hypertension. However, fruits and vegetables, especially, those that are consumed raw and or not properly washed, have been the major way for the transmission of human pathogens [3–5].

Intestinal parasitic infection may be acquired in different ways like by consumption of contaminated fruits, vegetables, other food stuff, and water [6]. Eating unclean, raw, or undercooked fruits and vegetables is one of the means by which the transmission of intestinal parasitic infections is propagated [7]. Fruits and vegetables act as vehicles for the transmission of parasitic infections when contaminated as a result of various associated factors related to planting, such as while they are still on the field, harvesting, transportation, storage, market chain, and even at home [5, 8].

Despite the fact that intestinal parasitosis is common in Jimma Town [9, 10], there are no studies conducted to assess the level of contamination of fruits and vegetables with parasites of medical and zoonotic importance. If our target is to control the intestinal parasitic diseases, it is not enough to depend merely on the chemotherapeutic intervention of identified cases, but need the concerted effort to reduce and eliminate the potential sources of infection. To our knowledge there is no published document to attest the level of parasitological contamination of fruits and vegetables in Jimma Town. Therefore, this study was designed to determine the level of parasitic contamination of selected fruits and vegetables and associated factors in Jimma Town.

## 2. Materials and Methods

### 2.1. Study Area and Period

The study was conducted in Jimma Town, which is located at south west of Ethiopia, about 352 km from Addis Ababa, the capital of Ethiopia. A cross-sectional study was conducted to determine the level of parasitic contamination of fruits and vegetables sold in selected local markets in Jimma Town from April 22 to May 14, 2013.

### 2.2. Sample Collection and Analysis

Eight types of fruits and vegetables including lettuce, cabbage, carrot, tomato, green pepper, banana, mango, and salad were purchased from four conveniently selected local markets, namely, “Bishishe,” “Hirmata Merkato,” “Kochi,” and “Agip” found in Jimma Town. Equal numbers of samples (45 each, totally 360 samples) were collected from the selected markets. The samples were collected, put in plastic bags, properly labeled, and brought to the Medical Parasitology Laboratory of Jimma University, for parasitological analysis.

A portion (200 g) of each fruit and vegetable was washed separately in 500 mL of normal saline for detaching the parasitic stages (ova, larvae, cysts, and oocysts) of helminths and protozoan parasites commonly assumed to be associated with vegetable contamination. After overnight sedimentation of the washing solution, 15 mL of the sediment was then transferred to a centrifuge tube using sieve, to remove undesirable matters. For concentrating the parasitic stages, the tube was centrifuged at 3000 rpm for five minutes [5]. After centrifugation, the supernatant was decanted carefully without shaking. Then the sediment was agitated gently by hand for redistributing the parasitic stages. Finally, the sediment was examined under a light microscope using ×10 and ×40 objectives. Modified Zeihl-Neelsen staining technique was also used for identification of oocysts of* Cryptosporidium* and* Cyclospora spp* as described elsewhere [11].

## 3. Data Analysis

Data were entered into, cleaned, and analyzed using SPSS for windows version 16.0. The difference between prevalence of intestinal parasites among different categories was compared using Pearson chi-square test. Univariate and multivariate logistic regression was used to identify factors associated with parasitic contamination of the fruits and vegetables. A *P value* of <0.05 was used as a statistically significant difference.

### 3.1. Ethical Issue

Ethical clearance was obtained from Jimma University Ethical Review Board. Data collection using questionnaire was done after the purpose of the study was explained to the respondents (vendors of fruits and vegetables) and verbal consent was obtained.

## 4. Results

A total of 360 samples of fruits and vegetables were collected from the local markets and examined for parasitological contamination. The results of the study showed that 208 samples were identified to be contaminated with at least one type of parasite, which gave rise to the overall contamination rate of 57.8%. These include 53% of green pepper, 68.9% of cabbage, 55.6% of lettuce, 77.8% of salad, 62.2% of carrot, 46.7% of tomato, 51.1% of banana, and 46.7% of mango (Table 1).

The stages and species of parasites detected include larvae of* Strongyloides* like parasite, ova of* Ascaris lumbricoides*,* Toxocara spp*,* Hymenolepis nana*,* and Hymenolepis diminuta*, oocysts of* Cyclospora spp* and* Cryptosporidium spp*, and cysts of* Giardia lamblia*, and* Entamoeba histolytica/dispar*.  Table 2 shows that* Strongyloides* like parasite (21.9%) was the most frequently detected parasitic contaminant followed by* Toxocara Spp* (14.7%),* Cryptosporidium Spp* (12.8%),* H. nana* (8.3%),* G. lamblia* (7.5%),* A. lumbricoides* (6.7%),* E. histolytica/dispar* (5.3%),* Cyclospora spp* (5.0%), and* H. diminuta* (1.4%).

The highest frequency of* Strongyloides* like parasite was detected in samples of salad and the least frequency from samples of carrot and tomato. Ova of* Toxocara Spp* was detected most frequently from cabbage samples but not recovered from mango samples. Ova of* A. lumbricoides* was detected from salad samples with highest frequency and not detected from samples of green pepper. Ova of* H. diminuta* were recovered from cabbage and carrot samples only.

Polyparasitic contamination was observed in fruits and vegetables examined in this study. 37.5% of the total samples were contaminated with two species of parasites, while 6.25% of the samples with three species of parasites and quadruple parasitic contamination were observed in two samples (Table 1).* Strongyloides* like parasite and* Toxocara* spp combination was the most frequently encountered with 35.48% of the multiple contamination.

The parasitic contamination rate among the different fruits and vegetables was significantly different (*P* = 0.027) Table 3. The highest prevalence of intestinal parasites was recorded in salad (16.8%) followed by cabbage (14.9%), carrot (13.5%), lettuce (12%), green pepper (11.5%), banana (11.1%), and tomato and mango each 10.1%. Further analysis with a binary logistic regression showed that, as-compared to mango, salad was significantly contaminated (AOR = 4.5, 95% CI (1.7, 11.9)); see Table 4.

In addition to the parasitological investigations, factors associated with contamination of fruits and vegetables were also assessed. These factors were assessed by interviewing the vendors of fruits and vegetables in the selected markets of Jimma Town. The educational status of the vendors was ascertained and the majority (52%) of the vendors had no formal education, while 36% of the vendors had primary education and only 12% had secondary education. There was no significant association between education level of vendors and parasitic contamination rate of the produces they were selling (*P* = 0.845), Table 3.

The samples were collected from four different local markets in Jimma Town. The results of the study showed that samples collected from “Hirmata Merkato” (29.8%) had high contamination rate followed by samples collected from “Bishishe” (28.4%), “Agip” (22.1%), and “Kochi” (19.7%) markets. The percentage contamination rate was significantly different among samples collected from the different markets (*P* = 0.003), Table 3. Samples were collected from both open markets and groceries. Samples from groceries contributed for 15.4% of the positive samples while 84.6% of the contamination was contributed by open markets with no statistically significant difference (*P* = 0.811), Table 3.

Another factor associated with parasitic contamination of fruits and vegetables is the act of washing the produces before displaying for sale. According to this study, majority (73.1%) of the produces were not washed before display for selling while only 26.9% of the produces were washed before displaying for sale. 82.2% of the unwashed produces were contaminated with one or more parasites, while 17.8% of the washed produces were contaminated with intestinal parasites. The cross tabulation of washing the produces before display for sale and result of parasitological analysis showed a significant difference in contamination rate among washed and unwashed produces (*P* = 0.000), Table 3. As compared to the washed produces, the odds of parasitic contamination for unwashed ones was 3.33 times (AOR = 3.3, 95% CI (1.9, 5.6)), Table 4.

The sources of water used for washing the produces among the vendors include pipe water (62.5%), well water (30.2%), and river water (7.3%). 35%, 48.3%, and 28.6% of produces washed by pipe water, well water, and river water were contaminated with at least one parasitic species, respectively. There was no significant difference in contamination rate among produces washed by water from different sources (*P* = 0.412), Table 3.

The means of display for selling is also another factor assessed for association with parasitic contamination of fruits and vegetables. Various means of display were observed among the vendors as follows: 67.5% of the produces are displayed on the floor by the road sides while 23.1% on tables by the road sides.

## 5. Discussion

The detection of intestinal parasitic stages from fruits and vegetables is an indicative of the fecal contamination from human and or animal origin. As in many tropical countries, intestinal parasites are widely distributed in Ethiopia not only due to the favorable climatic conditions for the survival and dissemination of the parasites but also due to the unsanitary conditions that facilitate fecal pollution of water, food stuffs, and soil [8].

The present study has attempted to assess the level of contamination and prevalence of different intestinal parasites from different fruits and vegetables sold in selected markets of Jimma Town. The overall parasitic contamination rate was found to be 57.8%, which is in agreement with the findings reported elsewhere [3, 5]. However, it is higher than what was reported in similar studies from other areas [8, 12–17]. On the other hand, it is lower when compared with the findings of some studies [18, 19].

The discrepancy between the present study and previous studies might be as a result of the variations in geographical locations, climatic and environmental conditions, the kind of sample and sample size examined, the sampling techniques, methods used for detection of the intestinal parasites, and socioeconomic status. So long as these factors differ, consequently the discrepancy of the results would be expected.

Salad (77.8%) was found to be the most frequently contaminated produce followed by cabbage (68.9%), carrot (62.2%), lettuce (55.6%), green pepper (53.3%), and banana (51.1%). Tomato (46.7%) and mango (46.7%) were the least contaminated. This variation among the produces might be due to the fact that salad, cabbage, carrot, and lettuce have uneven surfaces which make the parasitic stages attach more easily to the surface of these vegetables. The smooth surface of green pepper, tomato, and mango might reduce the rate of parasitic attachment hence had lower contamination rate [15].

In this study, Larvae of* Strongyloides* like parasite was the most frequently detected parasite with a prevalence of 21.9%. This might be due to the fact that the parasite has a free living state and does not require a host for its proliferation, in addition to its parasitic mode of life [5]. The predominance of* Strongyloides* like parasite is similar with similar studies conducted elsewhere [5, 8, 12, 17]. However, the finding is in contrast with what was reported by other investigators where* Ascaris lumbricoides*,* Cryptosporidium spp*,* E. histolytica/dispar*, and* Toxocara* spp were the predominant parasites detected [13–16].

Ova of* Toxocara* spp was the second most prevalent contaminant next to* Strongyloides* like parasite. This dominance might be attributed to the high fertility of* Toxocara* female adult producing up to 10,000 eggs daily and the resistant nature of the eggs, which may survive for up to ten years resisting harsh conditions in the environment [20].

*Cryptosporidium* spp was the third most frequently detected parasite in this study with a prevalence of 12.8%. This finding is lower than the finding of 29.3% reported from Alexandria, Egypt [15]. However, majority of researches did not report the parasite as vegetable contaminant [3, 5, 12, 13, 20–22]. This might be due to differences in methods used between this study and the previous ones. In the present study, modified acid fast stain was used for detection of the coccidian oocysts, while the majority of the previous studies did not.

No ova of hookworm species were detected from the samples examined in the present study. This is in agreement with other studies conducted elsewhere [3, 14, 16, 20, 23]. This might be due to the fact that hookworms have very short life span in the soil [24]. However others have reported the contamination of vegetables with hookworm species [5, 6, 25]. The differences might be attributed to differences in geographical locations, climate conditions, and the type of soil [26].

Multiple species contamination was observed in all kinds of produces examined in this study. This might indicate the possibility of high level contamination of the fruits and vegetables, which perhaps results in multiple parasitic infections in human. It might also indicate the persistence of intestinal parasitic infection in the area [5].

The contamination rate was significantly different for the samples collected from the different markets in which samples collected from “Hirmata Merkato” showed higher rate. This might be associated with the act of washing of the produces before display; 77.8% of the samples collected from the market were not washed.

Majority (69%) of the produces were displayed for sale on the floor where it is exposed to dusts and flies. It is well established that the flies can act as vectors for a number of pathogenic microorganisms including parasites like* Cryptosporidium parvum* and* Toxoplasma gondii* [27]. Besides, there might be bacterial and viral contamination of the produces during display for sale on the floor.

The habit of eating raw vegetables like salad and tomato is commonly practiced in the study area. Hence, the findings of the present study are of public health importance, requiring an appropriate intervention to prevent transmission of parasitic diseases that can be acquired through consumption of contaminated fruits and vegetables. However, this study did not address the effect of seasonal variation on the contamination of the fruits and vegetables. The findings of this study could not underscore the infectivity of the parasitic stages detected as viability study was not conducted.

## 6. Conclusion

In conclusion, this study highlighted the importance of raw fruits and vegetables as the potential source of transmission for intestinal parasites to humans. The fruits and vegetables contamination with the pathogenic parasites poses health risk to the consumers if consumed without proper cleaning and or cooking.

Prevention of contamination remains the most effective way of reducing food borne parasitic infection. A comprehensive health education should be given to vendors and farmers of fruits and vegetables and to the general population on the health risks associated with consumption of contaminated fruits and vegetables. The consumers should always observe the basic principle of food and personal hygiene, that is, thorough washing of the fruits and vegetables before eating and washing hands before meal.

The vendors of fruits and vegetables should avoid the contact of the produces with soil while display for selling. Further studies should be conducted on the viability of parasitic contaminants of fruits and vegetables. Also, other researches must be done to evaluate the level of parasitic contamination of farm produces, water, and soil in which fruits and vegetables are cultivated. These studies should also be conducted in different regions of the country.

## Figures and Tables

**Table 1:** Frequency distribution of polyparasitic contaminations among fruits and vegetables sold in selected markets of Jimma Town from April 22 to May 14, 2013.

Kind of produce	Number examined	Number positive (%)	Number of parasitic spp detected			
			One	Two	Three	Four
Green pepper	45	24 (53.3)	18 (40)	5 (11.1)	1 (2.2)	0
Cabbage	45	31 (68.9)	13 (28.8)	13 (28.8)	4 (8.9)	1 (2.2)
Lettuce	45	25 (55.6)	15 (33.3)	9 (20)	1 (2.2)	0
Salad	45	35 (77.8)	17 (37.8)	14 (31.1)	3 (6.7)	1 (2.2)
Carrot	45	28 (62.2)	12 (26.7)	13 (28.8)	3 (6.7)	0
Tomato	45	21 (46.7)	16 (35.6)	5 (11.1)	0	0
Banana	45	23 (51.1)	13 (28.8)	10 (22.2)	0	0
Mango	45	21 (46.7)	11 (24.4)	9 (20)	1 (2.2)	0

Total	360	208 (57.8)	115 (31.9)	78 (21.7)	13 (3.6)	2 (0.6)

**Table 2:** Prevalence of intestinal parasites in selected fruits and vegetables sold at selected markets in Jimma Town from April 22 to May 14, 2013.

Detected parasite	Frequency	Prevalence
*A. lumbricoides *	24	6.7%
*Cryptosporidium *Spp	46	12.8%
*Cyclospora *spp	18	5.0%
*E. histolytica/dispar *	19	5.3%
*G. lamblia *	27	7.5%
*H. diminuta *	5	1.4%
*H. nana *	30	8.3%
*Strongyloides *like parasite	79	21.9%
*Toxocara *Spp	53	14.7%

Total sample (*n* = 360)		

**Table 3:** Chi-square test of factors associated with parasitic contamination of fruits and vegetables sold in selected markets of Jimma Town from April 22 to May 14, 2013.

Variables	Result of parasitological analysis		*χ* ^2^	*P* value
	Pos (%)	Total		
Market				
HirmataMerkato	62 (68.9)	90	13.937	0.003
Bishishe	59 (65.6)	90		
Agip	46 (51.1)	90		
Kochi	41 (45.6)	90		
Total	**208** (**57.8**)	**360**		
Kind of produce				
Salad	35 (77.8)	45	15.85	0.027
Green pepper	24 (53.3)	45		
Cabbage	31 (68.9)	45		
Lettuce	25 (55.6)	45		
Carrot	28 (62.2)	45		
Tomato	21 (46.7)	45		
Banana	23 (51.1)	45		
Mango	21 (46.7)	45		
Total	**208** (**57.8**)	**360**		
Sources of the produces				
Farmers	56 (62.2)	90	2.723	0.256
Middle men	139 (57.7)	241		
Private garden	13 (44.8)	29		
Total	**208** (**57.8**)	**360**		
Market type				
Grocery	32 (59.3)	54	0.057	0.811
Open market	176 (57.5)	306		
Total	**208** (**57.8**)	**360**		
Washed before display				
Yes	37 (38.1)	97	20.980	0.000
No	171 (65)	263		
Total	**208** (**57.8**)	**360**		
Water source for washing purpose				
Pipe water	21 (35%)	60	1.772	0.412
Well water	14 (48.3)	29		
River water	2 (28.6)	7		
Total	**37** (**38.5**)	**96**		
Means of display				
On the floor	146 (58.6)	249	0.870	0.833
On shelf in shop	9 (60)	15		
On tables	47 (56.6)	83		
On wheel barrow	6 (46.2)	13		
Total	**208** (**57.8**)	**360**		
Handled by vendor who has				
No formal education	109 (58.6)	186	0.336	0.845
Primary education	82 (57.7)	142		
Secondary education	17 (53.1)	32		
Total	**208** (**57.8**)	**360**		

**Table 4:** Binary logistic regression of factors associated with parasitic contamination of fruits and vegetables sold in selected markets of Jimma Town from April 22 to May 14, 2013.

Variables	Laboratory result for parasitic contamination		
	Pos (%)	COR (95% CI)	AOR (95% CI)
Vegetable type			
Green pepper	24 (53.3)	1.3 (0.6, 2.9)	1.2 (0.5, 3.1)
Cabbage	31 (68.9)	2.5 (1.1, 5.9)∗	1.8 (0.7, 4.5)
Lettuce	25 (55.6)	1.4 (0.6, 3.3)	1.5 (0.6, 3.8)
Salad	35 (77.8)	4.0 (1.6, 9.9)∗	4.5 (1.7, 11.9)∗
Carrot	28 (62.2)	1.8 (0.8, 4.3)	1.8 (0.7, 4.4)
Tomato	21 (46.7)	1.0 (0.4, 2.3)	1.0 (0.4, 2.4)
Banana	23 (51.1)	1.2 (0.5, 2.7)	1.2 (0.4, 3.0)
Mango∗	21 (46.7)		
Washed before display			
No	171 (65)	3.0 (1.8, 4.8)∗	3.3 (1.9, 5.6)∗
Yes ∗∗	37 (38.1)		
Market			
Agip	46 (51.1)	0.5 (0.3, 1.0)	0.5 (0.2, 1.1)
Kochi	41 (45.6)	0.4 (0.2, 0.8)∗	0.4 (0.2, 0.8)∗
Hirmata Merkato	62 (68.9)	1.1 (0.6, 2.1)	1.1 (0.5, 2.0)
Bishishe∗∗	59 (65.6)		
Means of display			
On the floor	146 (58.6)	1.6 (0.5, 5.1)	1.9 (0.5, 6.8)
On shelf in shop	9 (60)	1.7 (0.3, 7.8)	1.3 (0.2, 7.3)
On tables	47 (56.6)	1.5 (0.4, 4.9)	1.8 (0.4, 6.9)
On wheel barrow∗∗	6 (46.2)		
Market type			
Grocery	32 (59.3)	1.1 (0.5, 1.9)	1.1 (0.5, 2.1)
Open market∗∗	176 (57.5)		
Handled by vendor who has			
No formal education	109 (58.6)	1.2 (0.6, 2.6)	1.4 (0.6, 3.1)
Primary education	82 (57.7)	1.2 (0.5, 2.6)	1.3 (0.5, 2.9)
Secondary education∗∗	17 (53.1)		

^*^Significant at *P* value of 0.05, ^**^Reference category, COR-crude odds ratio, AOR-adjusted odds ratio.
